# Three more steps toward better science

**DOI:** 10.12688/f1000research.16358.2

**Published:** 2019-04-01

**Authors:** Jose D. Perezgonzalez

**Affiliations:** 1 School of Aviation, Massey Business School, Massey University, Palmerston North, 4442, New Zealand

**Keywords:** philosophy of science, methodology, statistics

## Abstract

Science has striven to do better since its inception and has given us good philosophies, methodologies and statistical tools that, in their own way, do reasonably well for purpose. Unfortunately, progress has also been marred by historical clashes among perspectives, typically between frequentists and Bayesians, leading to troubles such as the current reproducibility crises. Here I wish to propose that science could do better with more resilient structures, more useful methodological tutorials, and clearer signaling regarding how much we can trust what it produces.

Science has striven to do better since its inception. For example, empiricism was sought as an alternative mode of learning as early as the XVI Century ( Ball, 2012); XIX Century researchers sought a less subjective approach to learning from data via frequentist statistics, which progressively displaced Bayesian inference ( Gigerenzer *et al*., 1989); in the XX Century, seeking a better way of establishing causation, Fisher (e.g., 1954) popularized a consistent framework of experimental design and frequentist inference based on small samples; Neyman & Pearson (e.g., 1928) expanded on Fisher’s statistical innovations to bring about more control of research power; Jeffreys (e.g., 1961) countered with a more nuanced approach toward evidential support for hypotheses via his Bayes factor; Cohen (1988) veered the focus away from significance testing and toward practical importance with his seminal work on effect sizes and power analyses; Mayo (e.g., 2018) is nowadays popularizing a framework based on severity testing for better frequentist inference; and computational advancements are giving full Bayesian inference a new opportunity to claw back the territory lost since the XX Century ( McGrayne, 2012).

Such historical drive has given us good tools for purpose, including philosophies and methodologies, as well as statistical tools for exploratory data analyses, data testing, hypothesis testing, and replication research. The path has not been easy, with a lot of effort gone onto warring among different philosophies, methodologies, and statistical approaches, and leading to troubles such as the current reproducibility crises (e.g., Fanelli, 2018).

Still, most approaches have been put forth and defended on the common goal of bettering science and, in their own way, all do so reasonably well. For example, Table 1 summarizes results obtained using different testing approaches, all concluding with similar inferences. Therefore, the real “enemy” is not what makes for better science but what makes for worse science: namely, problems with methodological control, with the misunderstanding and misuse of statistics, and with unsupported conclusions (i.e., with ethical concerns and with the use of scientific methods in a pseudoscientific manner; Perezgonzalez & Frías-Navarro, 2018).

**Table 1.** Reasonable conclusions based on frequentist and Bayesian results.

Case	Cohen’s d	Test	p	Decision	SEV	BF	Evidence
I (2t)	0.20	t _(44)_ = 0.67	0.507	H _0_	0.75	BF _01_ = 2.85	M _0_ = anecdotal
II (1t)	0.80	t _(44)_ = 2.71	0.995	H _0_	0.99	BF _01_ = 10.96	M _0_ = strong
III (2t)	0.80	t _(44)_ = 2.71	0.010	noH _0_	0.99	BF _10_ = 5.04	M _1_ = moderate
IV (1t)	-0.67	t _(31)_ = -1.88	0.965	H _0_	0.99	BF _01_ = 7.20	M _0_ = moderate
V (2t)	-0.67	t _(31)_ = -1.88	0.071	H _0_	0.51	BF _10_ = 1.25	M _1_ = anecdotal
VI (1t)	-0.93	t _(44)_ = -3.14	0.999	H _0_	0.99	BF _01_ = 12.08	M _0_ = strong
VII (2t)	-0.93	t _(44)_ = -3.14	0.003	noH _0_	0.99	BF _10_ = 12.70	M _1_ = strong

*Notes*. Based on data from ( Vincent, 2018; Perezgonzalez & Vincent, 2019). **Case**: tests are one-tailed (1t) or two-tailed (2t). **Cohen’s *d***: exploratory tests assessing observed effect sizes against Cohen *d* = 0.5 (i.e., the sample size—n1= 23; n2 = 23—was sensitive to *d* ≥ 0.5, one-tailed; Perezgonzalez, 2017). **Test**: *t*-tests statistics and degrees of freedom. **p**: *p*-values from independent *t*-tests (Fisher’s approach, e.g., 1954). **Decision**: frequentist decision—noH _0_ = reject H _0_; H _0_ = no decision—based on level of significance = 0.05 (e.g., Perezgonzalez, 2015). **SEV**: severity tests based on the observed effects (severity is strong if greater than 0.80; e.g., Mayo, 1996). **BF**: Bayes Factors with alternative model based on a Cauchy distribution (e.g., Rouder *et al*., 2009). **Evidence**: Bayesian evidence in favor of the null model (M _0_) or the alternative model (M _1_; e.g., Wagenmakers *et al*., 2018). The effect sizes of Cases II, IV, and VI had signs opposite to those expected (therefore, the high *p*’s); Cases III, V, and VII are two-tailed tests of Cases II, IV, and VI (thus, the similar *d*’s). Only Case V may lead a Jeffreysian to an inference contrary to those of frequentists; most likely, they would refrain from inferring support based on anecdotal posterior probabilities (e.g., Jarosz & Wiley, 2014).

Such enemy will be difficult to defeat. On first impression, science seems to suffer the fate of the ‘tragedy of the commons’, the ‘free-rider dilemma’ being, perhaps, its most specific affliction ( Fisher, 2008). A recent book by Taleb (2018) on asymmetry sheds some light on the gaming element of science, namely on its misuse of analytical models, agency problems, asymmetric information sharing, and the rationality of the enterprise. Taleb also proposes three solutions that we could expand upon to provide a synergic path for how to go about bettering science ( Perezgonzalez, 2018).

Firstly, there is a need to make ‘scientific structures’ more resilient, for them to deliver the outcomes they were set up for: widespread accessibility and quality control. For example, open access publishing is nowadays countering the paywall limitations of traditional scientific publishing and its bias toward novel research with significant results, thus addressing important academic and social backlashes ( Kelly, 2018; Schiltz, 2018). Unfortunately, it has also motivated the rise of predatory journals catering for the same pool of conscientious researchers. To counter the explosion of these predatory journals some idiosyncratic blacklists (e.g., the defunct Beall’s list) and organizational whitelists (e.g., Directory of Open Access Journals) have been created, albeit with mix success. Meanwhile, online repositories and preprint servers are challenging the entry costs of open access journals, thus making widespread communication more resilient but with the drawback of lacking good quality control—although overlay journals are taking care of the latter drawback.

Quality control itself has received more attention of lately, with some journals becoming more transparent about who peer-reviews, while platforms such as Publons.com provide peer-review services and credit, including access to peer-reviews when allowed. Among quality-control structures is worth mentioning F1000Research, a publication platform that sits at the fringe of a paid preprint and a fully transparent peer-reviewed open access journal. This seems a more resilient structure worthy of emulation and improvement.

Perhaps more importantly, a new need is becoming imperative: To find an effective solution to the indexing and curation of the ever expanding universe of research outputs. We do have, for example, Altmetric.com, albeit it is too geared toward scoring research outputs. Instead, what we need is an integrated solution to the indexing of both an output and all related content relevant to it, including post-publication reviews, comments in blogs and preprint servers, retraction notices, and the like. We also need a good solution to curating the entire spectrum of research outputs, moving from a plethora of stand-alone manuscripts toward mega-content organized as, for example, research topics.

Secondly, ‘minority movements’ do have an impact on science via creating the above new structures (e.g., open access, repositories…), but also by improving on legacy ones (e.g., post-publication review sites such as PubPeer.com). Paramount among such movements have been those calling for Open Science (e.g., Banks *et al*., 2018) and research ethics (e.g., Committee on Publications Ethics, RetractionWatch.com).

Minority movements also have an impact on other aspects of science, from calls toward a better use of frequentist statistics ( Perezgonzalez, 2015) to the outright banning of *p*-values ( Trafimow & Marks, 2015), to the alternative use of Bayesian statistics ( Wagenmakers *et al*., 2018b) or mixed approaches ( Perezgonzalez & Frías-Navarro, 2018). Because of the intrinsic social dynamics of minority groups, the polarization of inter-group attitudes and consequential external warring are not only unsurprising but also expected. Yet, as alternative scientific approaches mostly have a different research focus, science has been less productive than it could be because more effort has been put into warring among factions than into clearly explaining what each provides to the advancement of science ( Mayo, 2018). This has allowed specific methodological knowledge to be too much textbook-based, thereby more aligned with editorial concerns than with the advancement of science ( Gigerenzer, 2004), or to be polarized by the intrinsic dynamics of minority groups. Thus, what we presently need are good tutorials on the purpose of each approach and on how to effectively use them for such purpose; preferably, tutorials which are independently created by unfettered authors rather than centrally abridged by textbook editors, so as to provide a diversity of options able to address the same topic from different perspectives and to cater to different stakeholders (e.g., researchers, reviewers, and readers; novice and experts; technically-focused, philosophically-aware, as well as practitioners; etc.—see also the STRATOS Initiative, already working toward a similar goal, Stratos-initiative.org). Such diversity will also allow for progressively developing optimal tutorials that minimize steep learning curves, capture methodological errors, and avoid philosophical and interpretive misconceptions.

Finally, there is the need to signal how much ‘soul is in the game’ in each piece of published research. The pre-registration movement is achieving this via badges; most journals require authors to signal adherence to ethical principles via the corresponding disclaimers; some journals actively signal their peer-reviewing by naming peer-reviewers—e.g., Frontiersin.com, F1000Research.com—or by allowing open access to peer-reviews—e.g., via Publons.com. What we are presently lacking is good signaling to address methodological concerns and the avoidance of pseudoscience. That is, for authors to signal that they have followed, for example, Fisher’s approach to data testing, or Neyman-Pearson’s approach, or Mayo’s severity approach, or Jeffreys’s approach, or a full Bayesian approach; in brief, for them to signal when their research is compliant with the requisites of any of those approaches. The purpose of this signaling is to prevent what Farrington (1961, p. 311) already denounced, that “. . . there is no human knowledge which cannot lose its scientific character when [we] forget the conditions under which it originated, the questions which it answered, and the function it was created to serve”. This signaling could work in a manner similar to when authors specify a creative commons license for an open-access document: for a particular manuscript researchers could signal the specific methodological approach followed. This, of course, calls for negotiating the appropriate standards and for hosting them for quick referencing both by prospective authors and their peers.

In brief, following from the ideas of Taleb (2018), science could do better with more resilient structures, with more useful methodological tutorials, and with good signaling regarding how much we can trust what it produces. Thus my overall recommendation: let’s veer the focus from warring and onto improving our structures, tutorials and signals.

## Data availability

No data is associated with this article.
