# Supplementary material for: Three more steps toward better science
Source: F1000Res. 2019 Apr 1;7:1728. Originally published 2018 Oct 31. [Version 2] doi: 10.12688/f1000research.16358.2 (PMC6468710; doi:10.12688/f1000research.16358.2)
Supplement: Peer review history

# Peer review: Reviewer response for version 2

**DOI:** 10.5256/f1000research.20535.r46541

**Published:** 2019-04-25

**Authors:** Ben van Calster

**Affiliations:** 1 Department of Development and Regeneration, KU Leuven, Leuven, Belgium; 2 Department of Biomedical Data Sciences, Leiden University Medical Center, Leiden, The Netherlands

No further comments.

I have read this submission. I believe that I have an appropriate level of expertise to confirm that it is of an acceptable scientific standard.

# Peer review: Reviewer response for version 2

**DOI:** 10.5256/f1000research.20535.r46542

**Published:** 2019-04-15

**Authors:** Lincoln J. Colling

**Affiliations:** 1 Department of Psychology, University of Cambridge, Cambridge, UK

No further comments

I have read this submission. I believe that I have an appropriate level of expertise to confirm that it is of an acceptable scientific standard.

# Peer review: Reviewer response for version 1

**DOI:** 10.5256/f1000research.17868.r44358

**Published:** 2019-03-14

**Authors:** Lincoln J. Colling

**Affiliations:** 1 Department of Psychology, University of Cambridge, Cambridge, UK

I think this paper could benefit from a minor revision.  

 Some of the statements in the paper are a little vague. Most of the "meat" of the paper comes in the penultimate paper. It might be worth separating out and clearly stating the specific recommendations. 

 Table 1 presented SEV values. However, SEV is always with respect to a **specific inference** and an observation (an observation). For example, for an observation of e.g., mu = 17, the inference mu1 = 12 and the inference mu1 = 14 would have different SEV values, but Table 1 gives no indication of what inference is being made (I assume the inference is the same as the observed result, but this should at least be indicated somewhere). It also appears that Table 1 presented the results of t-tests. I would be inclined to include a test statistic (or an N or both - with an N the reader could calculate the test stat, or with a test stat the reader could calculate the N). I think it is almost essential, because the sample size is one of the key determinants of when the various inferential frameworks actually come apart. I think in its current form Table 1 paints a slightly misleading picture. 

 On Page 3, Para 1: It may be worth discussing "overlay journals". These exist in physics and computing, but recently an overlay journal in neuroscience has also been launch (Neuroscience, Behaviour, Data and Theory).

 Page 3, Para 5: There are some examples of these tutorial style papers: 1. Four reasons to prefer Bayesian analyses over significance testing, Dienes et al. ^1^ and 2. Statistical Inference and the Replication Crisis, Colling et al. ^2^.

I have read this submission. I believe that I have an appropriate level of expertise to confirm that it is of an acceptable scientific standard, however I have significant reservations, as outlined above.

## References

[1] DienesZMclatchieN: Four reasons to prefer Bayesian analyses over significance testing. *Psychon Bull Rev*.25(1) : 10.3758/s13423-017-1266-z 207-218 10.3758/s13423-017-1266-z 28353065PMC5862925

[2] CollingLSzűcsD: Statistical Inference and the Replication Crisis. *Review of Philosophy and Psychology*.2018; 10.1007/s13164-018-0421-4 10.1007/s13164-018-0421-4

# Response

**Published:** 2019-03-25

**Authors:** Jose Perezgonzalez

**Affiliations:** Massey University, New Zealand

Thank you very much for your review and useful pointers.

  

- *I think this paper could benefit from a minor revision. Some of the statements in the paper are a little vague. Most of the "meat" of the paper comes in the penultimate paper. It might be worth separating out and clearly stating the specific recommendations.*

I agree that the text is a little vague at times, albeit this is namely for the reason of lack of effective control on how the recommendations will eventually be implemented. For example, I can foresee the benefit of tutorials, in general (recommendation 2), but I cannot phantom whether there is a perfect tutorial to satisfy everyone (multiple and diverse tutorials may be needed for a “market-type” selection to kick-in, thus signalling helpful from less helpful ones). Actually, it is the third recommendation (in the penultimate paragraph) the one I see affected the least by variability in our imagination, as it calls for specific standards similar to standards already existing elsewhere (which is also why I placed it last, so to somewhat finish the commentary with a clearer, less vague, recommendation). It is for those reasons that I find it difficult to make the remaining recommendations more specific, and had to resort to the use of the conventional keywords “firstly”, “secondly”, and “finally” to somewhat constrain them to particular sections in the manuscript.

 Still so, I also aimed to put the recommendations more clearly towards the end of their sections: better indexing and curation as Recommendation 1 (this implies software-based indexing and curation, but I have little idea whether such software will work well); tutorials (but, again, I am not sure how well the idea will work; nonetheless, I added a correction linking to the STRATOS Initiative, at www.stratos-initiative.org, which may be one of the ways forward; other could be a methodology-based overlay journal, as you mentioned).

  

- *Table 1 presented SEV values. However, SEV is always with respect to a **specific inference **and an observation (an observation). For example, for an observation of e.g., mu = 17, the inference mu1 = 12 and the inference mu1 = 14 would have different SEV values, but Table 1 gives no indication of what inference is being made (I assume the inference is the same as the observed result, but this should at least be indicated somewhere). It also appears that Table 1 presented the results of t-tests. I would be inclined to include a test statistic (or an N or both - with an N the reader could calculate the test stat, or with a test stat the reader could calculate the N). I think it is almost essential, because the sample size is one of the key determinants of when the various inferential frameworks actually come apart. I think in its current form Table 1 paints a slightly misleading picture.*

I have added a new column with the *t*-test statistics and corresponding degrees of freedom. I have also extended the note on Severity to clarify it and also give a quick pointer for assessment, as *“SEV: severity tests based on the observed effects (severity is strong if greater than 0.80; e.g., Mayo, 1996)”*

  

- *On Page 3, Para 1: It may be worth discussing "overlay journals". These exist in physics and computing, but recently an overlay journal in neuroscience has also been launch (Neuroscience, Behaviour, Data and Theory).*

Thanks for the pointer. I wasn’t aware of overlay journals. But they certainly are a pretty good solution. I have added the following statement to paragraph 1, page 3: *“*—although overlay journals are taking care of the later drawback” *.*

  

- *Page 3, Para 5: There are some examples of these tutorial style papers: 1. Four reasons to prefer Bayesian analyses over significance testing, Dienes et al. and 2. Statistical Inference and the Replication Crisis, Colling et al.*

I am aware of several tutorials; I even have one of my own. However, I thought it would be not too good an idea to name them as the recommendation focuses on the idea of generating them; thus pointing to some (which may or may not be useful, in hindsight) seems distracting. I have, however, added a new entry to an initiative that seems to be working on the same idea, the STRATOS Initiative).

# Peer review: Reviewer response for version 1

**DOI:** 10.5256/f1000research.17868.r44361

**Published:** 2019-02-18

**Authors:** Ben van Calster

**Affiliations:** 1 Department of Development and Regeneration, KU Leuven, Leuven, Belgium; 2 Department of Biomedical Data Sciences, Leiden University Medical Center, Leiden, The Netherlands

This is an opinion piece, so I reviewed it as such. The paper discusses a few possible directions for improving the scientific process. I largely agree with the expressed opinions. That said, I think that the text remains quite general and vague at times. For example, with respect to the second issue, what do you mean with ‘tutorials which are independently created rather than centrally edited’? About the third issue: what is your specific suggestion? That researchers should better frame their work before starting it (as to what methodology and statistical approach they will use), and adhere to that when writing the report? This is unclear.

 Further comments:

- In the abstract, the statement ‘warring among different perspective’ may need a bit more clarity.
- The paragraph where the second issue is explained, could start with ‘Secondly’. (The first issue is introduced with ‘firstly’, the third issue with ‘finally’.)
- Table 1: too concise in my opinion. On what N are the p-valus based? Are the Cohen’s d values observed ones? A bit more information on severity tests might be useful. Perhaps columns that belong together (p and Decision; BF and Evidence; if I’m correct) may be put together more clearly.
- The paragraph starting with ‘such enemy will be difficult to defeat’ is quite vague. E.g. what do you mean with terms like ‘tragedy of the commons’, ‘asymmetric information sharing’, ‘rationality of the enterprise’, and others?
- Pre-print servers (line 2 of p3): do you refer to repositories like arXiv?
- Regarding scientific structures and peer-review: my issue is that peer review is very important (I am afraid that reviewers are sometimes the only people who check the contents of a study report), but it remains a largely uncredited – almost secret – endeavor. Don’t you agree that it still needs to be made more official, e.g. that universities do not only demand their researchers to publish papers, but also to deliver decent peer review reports? For example, when I started my tenure track, the university told me what they expected in terms of publication output, grants, and PhD guidance, but they said nothing about peer review, and they never evaluated me on these terms. A decent peer review should almost count as much as publishing a paper?
- 'Factions' (bottom of first column on p3): what is that?
- Would the STRATOS initiative ( www.stratos-initiative.org), coordinated by Willi Sauerbrei from Freiburg, be in line with the second issue? It brings together experts on different methodological topics in the context of observational studies, with the aim to provide guidance on how to address these topics. (I am a member of this initiative.)

I have read this submission. I believe that I have an appropriate level of expertise to confirm that it is of an acceptable scientific standard.

# Response

**Published:** 2019-03-25

**Authors:** Jose Perezgonzalez

**Affiliations:** Massey University, New Zealand

Thank you very much for you review (and apologies for the long response time).

 

- *This is an opinion piece, so I reviewed it as such. The paper discusses a few possible directions for improving the scientific process. I largely agree with the expressed opinions. That said, I think that the text remains quite general and vague at times. For example, with respect to the second issue, what do you mean with ‘tutorials which are independently created rather than centrally edited’? About the third issue: what is your specific suggestion? That researchers should better frame their work before starting it (as to what methodology and statistical approach they will use), and adhere to that when writing the report? This is unclear.*

I agree that the text is “general and vague at times”, albeit this is namely because I don’t know in which form the recommendations will eventually be implemented.

Independently created tutorials calls for multiple and diverse works to be done by independent authors or groups of authors, as opposed to them being ‘centrally edited’ by a publisher (e.g., of textbooks on methods, or statistics). The noted sentence follows from an earlier assertion, that “methodological knowledge   [is]   too   much   textbook-based,   thereby   more aligned  with  editorial  concerns  than  with  the  advancement  of science  (Gigerenzer,  2004)”. At the time, I also found it difficult to write it better without repeating ‘independently / independent authors’, and ‘centrally edited / textbook editors’. I have attempted it with synonyms here (albeit it may read a bit ‘forced’). It now reads “…tutorials which are independently created by unfettered authors rather than centrally abridged by textbook editors…”    

The third issue is a bit simpler than framing our work a priori (although it may help with this, as well). It is more like framing our work for publication. E.g., those who have followed a Fisherian approach, would say so but also write in a manner that is consistent with such approach, as the assumptions and inferential process are different to those following a Neyman-Pearson approach, or a Jeffreysian approach, etc. If no approach is clear or if they got mixed up in the process of doing the research, then no standard ought to be indicated (it would be misleading, otherwise).

The recommendation is more or less the following: In the same way we may decide to release a document under a particular creative commons license (or none), a license which we need to specify and which binds the document to it; we could also release a research report under a particular research standard (or none), and we shall specify such standard so that peer-reviewers can assess the manuscript as per compliance with those standards, and readers can understand the results in reference to such standards. This also means the standards need to be negotiated, approved and hosted as for facilitating quick referencing for the aforementioned peer-reviewers and readers (and, of course, authors).

- *Further comments:*
- *In the abstract, the statement ‘warring among different perspective’ may need a bit more clarity. The paragraph where the second issue is explained, could start with ‘Secondly’. (The first issue is introduced with ‘firstly’, the third issue with ‘finally’.)*

I have re- written the abstract, substituting ‘warring’ by “historical clashes among different perspectives, typically between frequentists and Bayesians”.

The second issue actually starts with ‘secondly’, when I introduced the idea of ‘minority movements’.

 

- *Table 1: too concise in my opinion. On what N are the p-valus based? Are the Cohen’s d values observed ones? A bit more information on severity tests might be useful. Perhaps columns that belong together (p and Decision; BF and Evidence; if I’m correct) may be put together more clearly.*

I have added a new column to Table 1, now describing the test, degrees of freedom, and test statistic. I also noted explicitly that Cohen’s *d* describes observed effects. The columns in the Table follow the suggested grouping:

- p-values and 'frequentist decision'
- Severity statistics (which is also a frequentist approach)
- Bayes factors and ‘Bayesian inference’

I also clarified a bit more severity testing.

- *The paragraph starting with ‘such enemy will be difficult to defeat’ is quite vague. E.g. what do you mean with terms like ‘tragedy of the commons’, ‘asymmetric information sharing’, ‘rationality of the enterprise’, and others?*

All those constructs are found in the book by Taleb (2018). The paragraph is meant to quickly brush over them as a quick introduction, as the really relevant constructs (also Taleb’s) follow: ‘scientific structures’, ‘minority movements’, and ‘soul in the game’.  

- *Pre-print servers (line 2 of p3): do you refer to repositories like arXiv?*

Both, actually, as they often have such a dual role: either as a final repository or as a repository of manuscripts prior to them being sent for publication elsewhere (nowadays, many journals accept the latter, as long as they are in repositories / preprint servers). I have nonetheless added the concept ‘online repositories’ to the text.

- *Regarding scientific structures and peer-review: my issue is that peer review is very important (I am afraid that reviewers are sometimes the only people who check the contents of a study report), but it remains a largely uncredited – almost secret – endeavor. Don’t you agree that it still needs to be made more official, e.g. that universities do not only demand their researchers to publish papers, but also to deliver decent peer review reports? For example, when I started my tenure track, the university told me what they expected in terms of publication output, grants, and PhD guidance, but they said nothing about peer review, and they never evaluated me on these terms. A decent peer review should almost count as much as publishing a paper?*

I fully agree. In fact, I think that a decent peer-review should count as much as publishing a paper and academics could, ideally, gain tenure and the like on peer-reviewing alone, not just on teaching or  researching, as this allows for good, committed peer-reviewers and increased quality standards (the problem is how to define ‘decent’!). That’s why I added an entry on ‘quality control’ under recommendation 1 for more resilient scientific structures. However, it will be very difficult to know who has peer-reviewed what and with what quality. This means that all comes down to trust: the academic on the tenure track would claim to have done ‘x’ reviews for ‘y’ journals…but it will be difficult to prove.

Thus far, I know of platforms such as Publons, PubPeer, FrontiersIn, and F1000Research, which would somewhat “reward” reviewers and/or publish peer-reviews. Of these, Publons actually rewards peer-reviewing and allows for generating some statistics in the form of percentiles and graphs. But reviews are only displayed depending on particular journal permissions. On the other hand, F1000Research does actually publish reviews and give them a doi. But there is no statistical summary of any form for reviewers.  Thus, independently acknowledging / rewarding peer-review will be difficult unless the action of peer-reviewing have been somewhat independently ‘vetted’ (Publons have such control…but I am not too sure how well it works) or peer reviews are openly displayed (F1000Research). I don’t’ see Universities caring for a career in peer-reviewing any time soon, though.  

- *'Factions' (bottom of first column on p3): what is that?*

Factions follow the “war” theme of Mayo’s latest book, consistent with similar concepts in the paragraph (enemies, polarization, warring, etc.). I have repeated Mayo’s reference in this paragraph.

- *Would the STRATOS initiative (www.stratos-initiative.org), coordinated by Willi Sauerbrei from Freiburg, be in line with the second issue? It brings together experts on different methodological topics in the context of observational studies, with the aim to provide guidance on how to address these topics. (I am a member of this initiative.)*

Yes… I think that you have almost ‘pulled the rug from under my feet’ here. The STRATOS initiative is pretty much in line with that point. I have added the following reference to the manuscript (“ *—see also the STRATOS Initiative, already working toward a similar goal, www.stratos-initiative.org*” ).
